# Acculturation of immigrant diet, basic taste responses and sodium appetite

**DOI:** 10.1017/jns.2018.12

**Published:** 2018-07-25

**Authors:** Micah Leshem, Haymanot Dessie-Navon

**Affiliations:** Psychology Department, University of Haifa, Haifa, 3498838, Israel

**Keywords:** Basic tastes, Dietary acculturation, Ethiopian immigrants, Lingual papillae, 6-*n*-Propylthiouracil (PROP), Salt appetite, Sex differences, EI, new Ethiopian immigrant, ES, veteran Ethiopian immigrant student, NS, native Israeli student, MANCOVA, multivariate ANCOVA, PROP, 6-*n*-propylthiouracil, VAS, visual analogue scale.

## Abstract

In young new Ethiopian immigrants (EI, about 0·5 years since immigration; *n* 20), veteran Ethiopian immigrant students (ES, about 13 years since immigration; *n* 30) and native Israeli students (NS; *n* 82), dietary macronutrients and electrolytes, and responses to basic tastes were compared in a cross-sectional design. From EI, to ES, to NS, dietary energy, protein, fat, and Na^+^ increase, whereas carbohydrates, K^+^ and Ca^2+^ do not differ. Corrected for energy intake, only Na^+^ increases. EI consume less dietary Na^+^, like foods with less Na^+^ content, salt their food less, yet show a greater hedonic response to salt taste. In contrast, preference for sweet does not differ. Taste psychophysics, 6-*n*-propylthiouracil (PROP) responses and lingual fungiform papillae density differ by group (and sex), but do not relate to dietary intake. Together, these changes could reflect dietary acculturation, increasing overall intake, Na^+^ in particular, accompanied by decreasing taste sensitivity, and changes in sensory perception and preference in these Ethiopian immigrants. The fact that immigrants find salt more hedonic, yet eat less of it, could suggest increased sensitivity to its taste, and might suggest restoring sensitivity to reduce Na^+^ intake for all. Similar alterations in taste sensory responses might be obtained in other forms of dietary flux. Understanding dietary acculturation can focus efforts (e.g. on Na^+^), to anticipate the disease burden of diets of affluence among immigrants. Yet, these immigrants’ nutrition is healthier in its low fat and Na^+^, suggesting that nutritional advice should focus on preservation, as well as prevention. Our study adds Ethiopian nutritional acculturation to that of the varied immigrant groups around the world.

Immigration often imposes far-reaching changes in nutrition. In particular, immigration to affluent societies with richer and more varied diets is often associated with increased energy, fat and Na^+^ intake, and compromised health. This includes diabetes, hypertension and obesity, which have been reported for Ethiopian immigrants to Israel; however, surprisingly, alteration in Na^+^ intake, which is implicated in these and other ailments, has been largely neglected^(^^1^^–^^12^^)^.

Changes in diet are accompanied by changes in preference, but there have been no studies of the sensory changes accompanying dietary acculturation^(^^13^^)^. Differences in taste responses have been related to sex, age, BMI, genetics (e.g. sensitivity to the taste of 6-*n*-propylthiouracil (PROP)) and lingual papilla density. These have been postulated to influence food choice, energy intake, and, ultimately, body weight and health, and thus may contribute to immigrant dietary acculturation and health^(^^14^^–^^26^^)^.

Here, in a cross-sectional design, we compared young, new (about 6 months) Ethiopian immigrants (EI), about 13 years veteran Ethiopian immigrant students (ES) and native Israeli students (NS), documenting differences in diet composition and taste responses, to suggest a longitudinal perspective on immigrant dietary and sensory acculturation. We evaluated dietary macronutrient and electrolyte intake, taste responses to intensity and hedonics of four basic tastes (NaCl, citric acid, sucrose and quinine HCl), PROP taster status and density of anterior lingual fungiform papilla (except in EI). Because of its relation to hypertension in immigrants^(^^1^^,^^10^^)^, we focused on salt appetite, the predilection for salt expressed in the variety of ways it is ingested.

## Materials and methods

### Participants

A total of eighty-two undergraduate NS and thirty undergraduate ES aged 18–33 years in the University of Haifa were recruited by posted notices. Twenty EI of similar age in an immigrant ‘absorption centre’^(^^27^^)^ agreed to participate. Availability of immigrants matching the group criteria constrained group size and sex ratio (Table 1).

**Table 1.** Demographics and taste characteristics (Mean values with their standard errors)

	New Ethiopian immigrants (*n* 17–20)		Veteran Ethiopian immigrant students (*n* 28–30)		Native Israeli students (*n* 76–82)	
	Mean	se	Mean	se	Mean	se
Age (years)	.20·6**	0·7	23·6	0·3	22·7	0·3
BMI (kg/m^2^)	20·8	0·4	21·3	0·6	22·8*	0·4
Women (*n*)‡	5		21		59	
Time since immigration (years)	0·48	0·04	13·13**	0·94	–	
Activity score	7·1	0·9	6·1	0·5	7·3	0·4
Cigarettes (no./d)	0·3	0·3	3·8†	1·4	2·2	0·6
Papilla density	–	–	12·3*	1·1	15·5	0·7
PROP	67·7*	8·4	59·4	5·6	50·2	3·1
Taster type (%)	12, 35, 53		13, 47, 40		13, 62, 24	

PROP, 6-*n*-propylthiouracil.

Different from unmarked groups: * *P* < 0·05, **P < 0·01.

† Different from new Ethiopian immigrants (*P* = 0·013).

‡ *P* < 0·001 (*χ*^2^ (2) = 16).

The university institutional review board provided ethics approval, and participants were advised to omit questions or tests they preferred. That, and missing data, varied sample sizes.

EI were not proficient in Hebrew, and were tested in Amharic, by Amharic-speaking researchers.

Participants were requested not to eat, drink (except water) or smoke for 1 h prior to the test session, which lasted some 90 min. At the end of the session weight and height were recorded.

Participants received credits or the equivalent of about $10 in shekels, by choice.

### Background questionnaire

The questionnaire queried age, dietary restrictions, smoking frequency, physical activity and phase of menstrual cycle. For EI, socio-economic status cannot be compared because it was determined by the absorption centre where they lived and received all basic requirements. ES and NS were identical in university educational level. Economic status (scholarships, parental income) was not queried.

### Dietary intake, seasoning, and liking questionnaire

Respondents reported the previous 7 d frequency and amount of consumption of some seventy-six items of food and drink (including items the participants added) covering the Israeli diet and available Ethiopian foods. This was analysed for dietary energy, macronutrient, electrolyte and water content. Each item was scored for liking on a five-level scale (−2 ‘dislike intensely’, 0 neutral, +2 ‘like very much’)^(^^28^^–^^30^^)^. Seasoning was scored on a four-level ordinal scale. Only salt and sugar are analysed here.

### Psychophysical assessment of hedonics and intensity of basic tastes

Intensity and hedonics were evaluated with oral sprays and visual analogue scales (VAS). Using perfume bottles, the experimenter sprayed a single pulse of 0·29 ml of each taste concentration in fixed and counterbalanced semi-randomised orders (excluding sequential concentrations) into each participant's mouth. Using VAS, participants rated (horizontal on-screen slider) each concentration for taste intensity (‘how strong is the taste?’, in Hebrew) anchored by ‘don't feel anything’ and ‘very strong’, and on a separate screen, hedonics (‘how tasty is it?’) anchored by ‘not tasty’ and ‘very tasty’^(^^28^^–^^30^^)^. For the new immigrants, instructions and questions were explained in Amharic.

Six concentrations of NaCl (2·5 mm–2·56 m by quadruple dilution steps), sucrose (0·1–27 % (w/v), treble steps), citric acid (1·2–280 mm, treble steps), and quinine HCl (0·01–0·35 mm, double steps) were tasted (Fig. 3). Participants rinsed their mouths between concentrations with distilled water. The order of taste testing was NaCl, HCl, sucrose and quinine (tested last to avoid carry-over effects). Taste tests were separated by 10 min of questionnaire.

Concentration–response served for the taste analyses, and the mean of the three highest concentrations (superscripted ^456^), as suprathreshold responses, served for correlations (Table 2, Figs 2, 3 and 4)^(^^28^^–^^30^^)^.

**Table 2.** Group comparisons (Descriptive means with their standard errors)

	New Ethiopian immigrants (*n* 18–20)		Veteran Ethiopian immigrant students (*n* 39–30)		Native Israeli students (*n* 80–81)	
	Mean	se	Mean	se	Mean	se
Adding salt†	1·05	0·15	2·12*	0·19	2·27*	0·23
Adding sugar†	0·65	0·09	0·70	0·08	0·65	0·07
Salt in soup (%)	1·3	0·2	1·3	0·1	1·2	0·1
Sucrose in tea (%)	8·9	1·1	10·2	0·8	10·6	0·5
Salt tidbits‡	7·10	1·42	15·53	2·95	15·69	1·94
Sweet tidbits‡	2·60**	0·61	7·07	1·67	11·12	1·56
Salt appetite§	49·1	2·3	65·5**	3·4	67·2**	2·2
Sweet preference§	63·7	3·1	66·2	3·9	63·6	2·6
Hedonics NaClǁ	48·9	6·0	31·2**	3·2	30·8***	1·9
Intensity NaClǁ	60·3	4·6	71·9	3·6	63·9	2·2
Hedonics sucroseǁ	68·1	3·3	68·4	4·0	60·6	1·7
Intensity sucroseǁ	61·8	3·3	70·9*	3·4	60·9	2·0
Hedonics citricǁ	121·8	20·2	81·6	10·5	95·7	7·1
Intensity citricǁ	40·6	6·7	27·2	3·5	31·9	2·4
Hedonics quinine HClǁ	14·3	3·8	18·3	2·6	24·9**	1·6
Intensity quinine HClǁ	67·0	6·9	67·3	4·0	60·7	2·5

Different from unmarked mean except where only one mean is indicated, which refers to the greatest mean difference: * *P* < 0·05, **P < 0·01, ***P < 0·001. Significance refers to adjusted values.

† Questionnaire score, 0–3.

‡ Number of tidbits eaten.

§ Sum of above, transformed scores, equally weighted, respectively for salty and sweet.

ǁ Mean of three highest concentrations (visual analogue scale).

As a cross-modal control to test whether instructions for the VAS were understood across groups, 6 2 cm squares of greyscale gradations between white and black, presented in random order, were scored for how ‘black’ they were (Fig. 2(c) and (f))^(^^29^^)^.

### Sweet and salty tidbits

After the taste tests, while completing the questionnaires, two familiar, commercial, salty (890 and 780 mg/100 g Na^+^) and sweet (120·5 and 146 mg/100 g Na^+^) snack items in bite-size tidbits on separate saucers were placed on the tabletop near the participants, who were invited to freely eat of them. The number and type of tidbits eaten was recorded discreetly^(^^28^^–^^30^^)^. A cup of water was also presented.

### Preferred concentration of NaCl in soup, and sugar in tea

The test is based on that used by Stone & Pangborn^(^^31^^)^. Tomato soup was prepared by diluting pure tomato paste concentrate (22°Bx; Sanlakol) with nine parts water. Participants were presented with two 100 ml cups of hot (about 45 ± 5°C) tomato soup, one unsalted and one with 3·0 % (w/v) NaCl. The word ‘salt’ was not used; the participants were asked to taste the ‘flavoured’ and the ‘unflavoured’ cup of soup, and then, using a 5 ml teaspoon, mix them into a third cup, into which the researcher had poured some of the unflavoured soup, until the soup was ‘most tasty’. The Na^+^ content of the mixture was determined. The procedure for determining the preferred concentration of sugar in tea was similar, with one cup containing unsweetened tea, and the other tea with 20 % (w/v) sucrose^(^^28^^–^^30^^)^.

### Evaluation of salt appetite

Dietary Na^+^ and salting, from the dietary questionnaire, hedonics of salt scored as the mean of the three highest concentrations of NaCl oral sprays, number of salty tidbits eaten, and preferred concentration of salt in soup were ranked and summed to provide an unweighted mean ‘salt appetite’ score. Analogous four measures for sweet (there is no measure of dietary ‘sweet’), provided the control ‘sweet preference’^(^^28^^,^^30^^,^^31^^)^.

### Biography of putative sodium loss

Dehydrational events during the lifetime of the participants were queried, and participants posted a questionnaire to their mothers querying the frequency, and severity, of nausea and vomiting by trimester while pregnant with the participant^(^^28^^–^^30^^,^^32^^–^^35^^)^. Most EI mothers were not available.

### 6-*n*-Propylthiouracil responses and taster type

Participants tasted a filter disc impregnated with 0·025 mg PROP (kindly donated by Linda Bartoshuk), scaled their sensation of intensity on a labelled magnitude scale, and were classified as super tasters, tasters or non-tasters^(^^36^^)^.

### Papilla density

Density of fungiform papillae in the anterior tongue was assessed using blue food colouring, and pressing to the tongue a plastic microscope slide with two glued 7 mm diameter binder punch-holes. The two punch-hole areas were centred 5·5 mm either side of the midline and photographed^(^^36^^,^^37^^)^. Counting was blind to group. Papilla density on the two sides correlated (0·898; *P* < 0·000), so their mean served for the analyses. Papillae were not counted in EI for whom the procedure was too invasive.

### Statistical methods

Based on a previous study^(^^29^^)^, power for EI–NS and EI–ES comparisons for dietary macronutrients exceeds 99 %, and for Na^+^, 94 and 82 %, respectively (unequal group sizes, 20/80 and 20/30, two-sided tests, 5 % significance level), suggesting the sample sizes are adequate for reasonable confidence in the dietary comparisons.

Multivariate (mixed between-within) ANCOVA (MANCOVA), or univariate analyses of covariance, adjusted for smoking (which can affect basic taste sensation^(^^38^^,^^39^^)^), age and BMI, because they differed significantly (Table 1), ANOVA, followed by the least significant difference (LSD) test for *post hoc* comparisons (general linear model), the Kruskall–Wallis test, as appropriate, and the Spearman correlation coefficient with Benjamini–Hochberg correction with *Q* = 0·25 were used. Probabilities over 0·05 (two-tailed) were not considered significant or detailed; the standard error of the mean is the measure of variability throughout (IBM SPSS 23 statistical package).

## Results

### Dietary intake, seasoning, and liking questionnaire

**Fig. 1.**
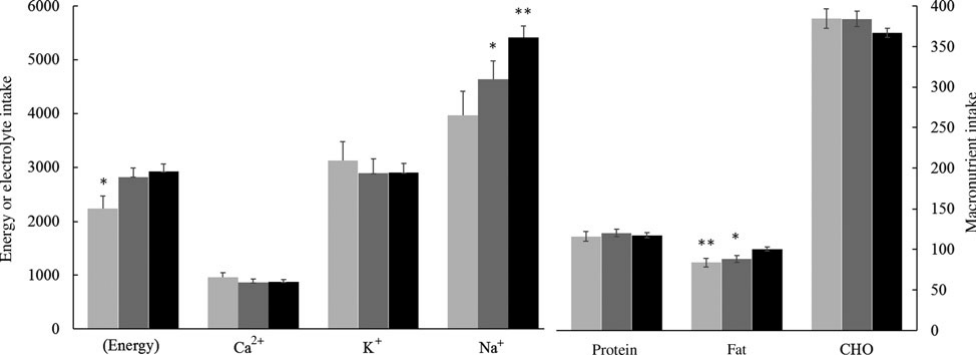
Dietary energy, macronutrients and electrolytes by group: new Ethiopian immigrants (░); veteran Ethiopian immigrant students (▒); native Israeli students (■). Values are means, with standard errors represented by vertical bars. Different from unmarked bars: * *P* < 0·05, ** *P* < 0·01. Adjusted for energy, only Na^+^ differs significantly between groups (see text). Electrolytes and energy are in mg and kcal (left axis); macronutrients are in g (right axis). To convert energy in kcal to kJ, multiply by 4·184. CHO, carbohydrate.

MANCOVA of seven dietary components (three macronutrients, three electrolytes, energy) by group and sex revealed an interaction of group and dietary nutrient (*F*(12,726) = 5·7; *P* < 0·000) and a group effect (*F*(2,121) = 6·6; *P* < 0·002). Subsequent univariate ANCOVA for individual nutrients showed group differences for dietary energy (*F*(2,121) = 4·3; *P* = 0·016), protein (*F*(2,121) = 3·4; *P* = 0·037), fat (*F*(2,122) = 7·1; *P* = 0·001) and Na^+^ (*F*(2,122) = 8·1; *P* = 0·001). Carbohydrates, Ca^2+^, K^+^, group and sex differences were not significant (Fig. 1).

Of the covariates, smoking increased dietary intake, specifically energy, fat, Na^+^ and alcohol (not detailed).

To further examine the group differences each macronutrient and electrolyte was analysed by group and sex controlling for energy and the other five dietary components (as well as age, smoking and BMI). A group effect was only found for Na^+^ (*F*(2,115) = 3·33; *P* = 0·039), suggesting that the increase in Na^+^ intake across groups was at least partially independent of other dietary components. *Post hoc* tests confirmed that EI ingested less Na^+^ than NS (*P* = 0·027; Fig. 1).

Adding salt, but not sugar, differed by group (*F*(2,118) = 4·4; *P* = 0·014), EI adding less salt than ES and NS (Table 2).

EI showed an inverse correlation between liking scores and Na^+^ content of seventy-two food items (*r*_s_ −0·395; *P* = 0·0009).

Alcohol intake did not differ by group: 21·7 % of men and 35·7 % of women drank no alcohol.

### Psychophysical assessment of intensity and hedonics of basic tastes

#### Intensity

**Fig. 2.**
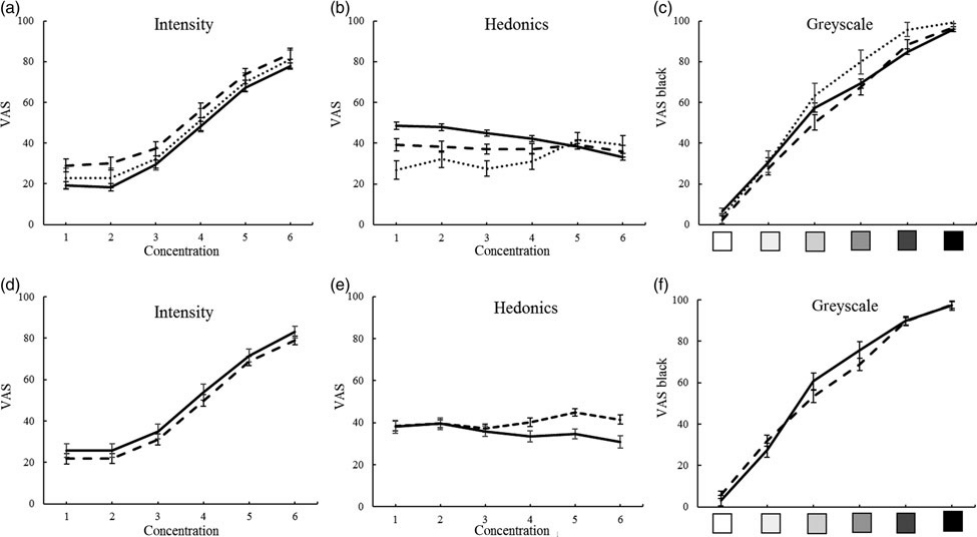
Overall responses to basic tastes and cross-modal greyscale control by group ((a), (b), (c); ···, new Ethiopian immigrants; ---, veteran Ethiopian immigrant students; ––, native Israeli students) and sex ((d), (e), (f); ---, men; ––, women). Significant effects for (a), (b) and (e) are detailed in the text. Values are means, with standard errors represented by vertical bars. VAS, visual analogue scale.

MANCOVA of the four tastes, six concentrations, three groups and sex produced significant interactions of taste with group, taste with sex, the three-way interaction, as well as concentration and taste and the three-way interaction with sex. The group effect (*F*(2,122) = 5·8; *P* = 0·004) suggests ES scoring greater intensity than NS (*P* < 0·001) (Fig. 2(a)).

Because taste figures in both significant interactions, each taste was examined separately (MANCOVA). Concentration is significant, as expected, and therefore not detailed.

**Fig. 3.**
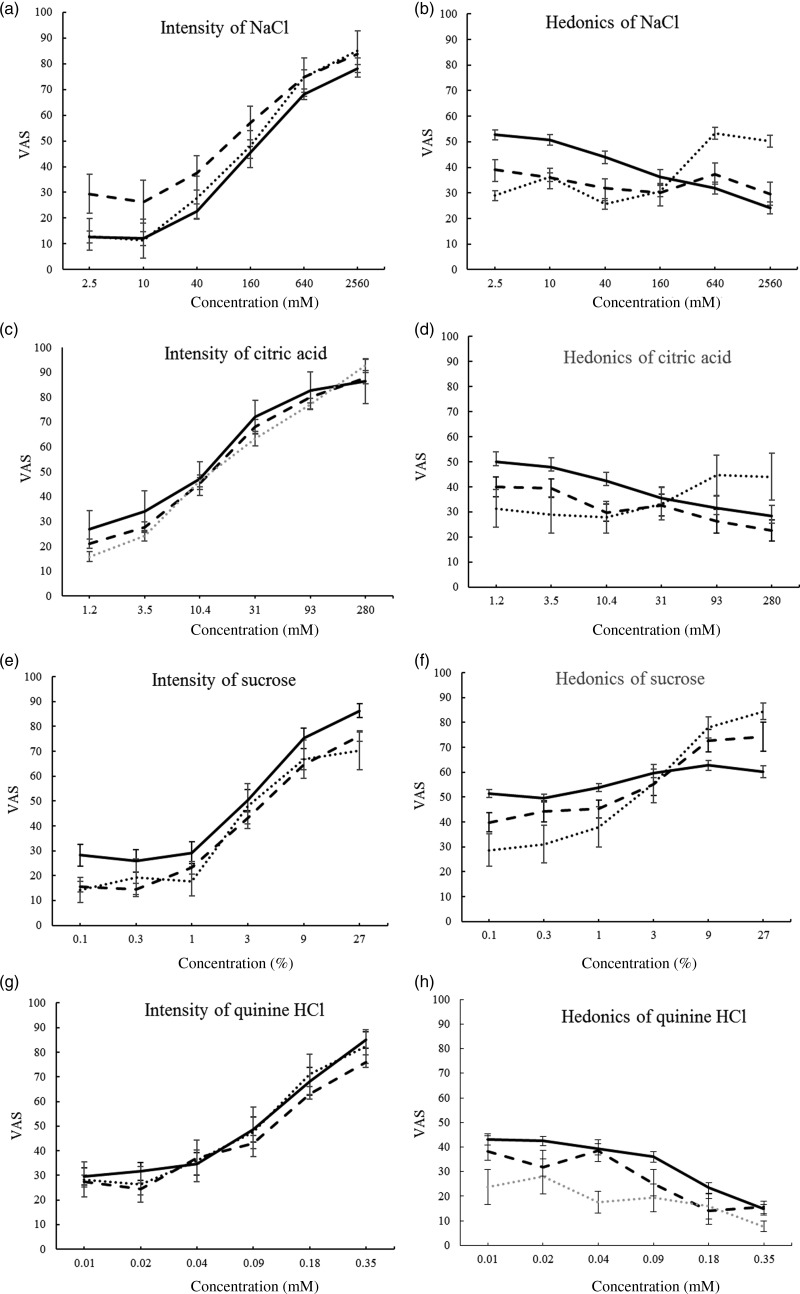
Intensity and hedonics of four basic tastes by group (···, new Ethiopian immigrants; ---, veteran Ethiopian immigrant students; ––, native Israeli students). There were significant effects for group and/or group interactions for all the graphs, which are detailed in the text. Values are means, with standard errors represented by vertical bars. VAS, visual analogue scale.

Intensity of salt solutions of six concentrations, three groups and sex reveals a group effect (*F*)2118) = 8·1; *P* = 0·001), with greater intensity in ES compared with NS and EI, respectively (*P* < 0·001 and 0·05) (Fig. 3(b)).

**Fig. 4.**
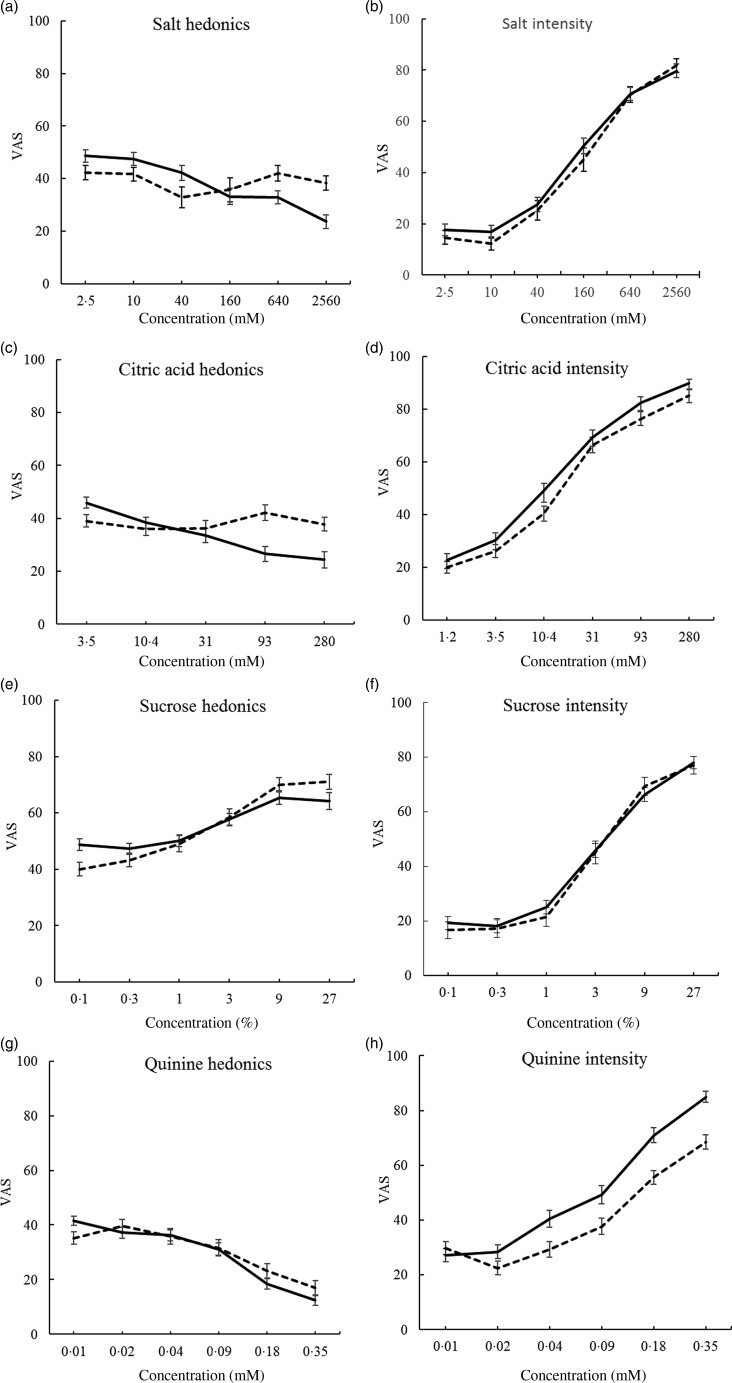
Intensity and hedonics of four basic tastes in men (---) and women (––). Significant effects and/or interactions for sex for (a), (c), (d) and (h) are detailed in the text. Values are means, with standard errors represented by vertical bars. VAS, visual analogue scale.

Citric acid intensity shows an interaction of group and sex (*F*(2,118) = 4·7; *P* = 0·011) (see Figs 3(d) and 4(d)), probably because NS women rate it more intense than NS men, with the opposite tendency in the other groups (not shown).

Sucrose intensity differed by group (*F*(2,118) = 6·8; *P* = 0·002), greater in ES compared with NS and EI, respectively (*P* < 0·001 and 0·05) (Fig. 3(f), Table 2).

Quinine HCl intensity differed by group (*F*(2,118) = 4·1; *P* = 0·02), with EI scoring greater intensity than NS (*P* = 0·012) (Fig. 3(h)). Women and men differed (*F*(1,118) = 12·7; *P* = 0·001), women rating VAS of quinine intensity as greater than men (58·0 (se 3·5) *v.* 41·0 (se 2·6)), and there was an interaction of sex and concentration (*F*(5,590) = 2·8; *P* = 0·018), because women scored concentrations as more intense than men (Fig. 4(h), Table 2).

#### Hedonics

MANCOVA of the four tastes, three groups, sex and six concentrations produced significant interactions of concentration with group, with sex and with taste, as well as concentration and taste and the three-way interactions with group and with sex, justifying the individual taste MANCOVA below. A main effect of group (*F*(2,121) = 6·9; *P* = 0·001), with NS rating greater hedonics than ES and EI, respectively (*P* = 0·021 and *P* < 0·002), is of unclear significance, occurring only in the lower concentrations which were barely distinguishable (Fig. 2(b)), and a main effect of sex (*F*(1,121) = 4·8; *P* = 0·03) appears due to greater aversion in women (Fig. 2(e)).

Hedonics of salt solutions showed interactions of concentration by group (*F*(10,585) = 3·9; *P* = 0·000) (Fig. 3(a)). An interaction of concentration and sex (*F*(5,585) = 4·5; *P* = 0·000) suggests that whereas women showed a dose-dependent aversion, men were less responsive to increases in concentration (Fig. 4(a)).

Citric acid hedonics differed by group (*F*(2,118) = 3·2; *P* = 0·045), with EI rating highest (Fig. 3(c)). An interaction of sex with concentration (*F*(5,590) = 4·6; *P* = 0·000) seemed due to higher rating by men, and dose-dependent aversion in women but not in men (Fig. 4(c)).

Sucrose hedonics showed an interaction of concentration and group (*F*(10,590) = 4·5; *P* = 0·000), although sucrose^456^ showed no significant effects (Fig. 3(e), Table 2).

Quinine HCl hedonics differed by group (*F*(2,118) = 8·7; *P* = 0·000), with EI most averse and NS least, EI differing from ES and NS (*P* = 0·014 and *P* = 0·000, respectively), and an interaction with concentration, (*F*(10,590) = 2·0; *P* = 0·035), possibly because EI showed a lesser dose–response (Fig. 3(g)). Women rated quinine more aversive than men (*F*(1,118) = 4·0; *P* = 0·049) (Fig. 4(g)).

#### Cross-modal control

ANOVA of the greyscale gradation VAS for the three groups, sex and six concentrations showed a three-way interaction (*F*(10,605) = 2·1; *P* = 0·024) (due to EI women scoring dark colours higher), but no significant effects of group or sex, suggesting that VAS is comparable across groups (Fig. 2(c) and (f)).

### Sweet and salty tidbits

MANCOVA (adjusted for age, BMI, smoking) for salty and sweet tidbits, by group and sex showed a significant group effect (*F*(4,242) = 2·9; *P* = 0·021), with different sweet tidbit intakes (*F*(2,121) = 5·3; *P* = 0·006) (Table 2).

### Preferred concentration of NaCl in soup, and sugar in tea

MANCOVA of soup and tea, group and sex revealed the expected difference in concentration of salt in soup and sugar in tea (not shown), with an effect of sex (*F*(1,122) = 6·9; *P* = 0·01), because women sweetened their tea less than men (8·6 (se 0·7) *v.* 11·2 (se 0·6) %).

### Evaluation of salt appetite

Salt appetite differed by group (corrected for age, smoking and BMI) (*F*(2,119) = 5·3; *P* = 0·006), lowest in EI and highest in NS. Sweet preference did not differ significantly between groups (Table 2).

### Biography of putative sodium loss

Lifetime tendencies for vomiting, diarrhoea, dehydration or their composite score did not correlate with salt appetite or sweet preference. The EI group did not have their mothers in the country to contact. ES mothers reported higher measures of vomiting and distress in the second and third trimesters than NS mothers (Kruskall–Wallis, *P* < 0·05, data not shown).

### 6-*n*-Propylthiouracil responses

ANOVA showed that PROP responses differed by group (*F*(2,123) = 5·0; *P* = 0·008) (Table 1), and were higher in women (*F*(1,123) = 5·0; *P* = 0·027; 71·1 (se 6·2) *v.* 53·7 (se 4·7)).

PROP responses correlated with intensity and hedonics of VAS for the mean three highest concentrations as follows:

For intensity of salt^456^, *r*_s_ 0·175, *P* = 0·049, *n* 127.

For intensity of sucrose^456^, *r*_s_ 0·171, *P* = 0·055, *n* 127, and hedonics for men, *r*_s_ 0·394, *P* = 0·007, *n* 46 (but inversely for NS women, *r*_s_ −0·366, *P* = 0·005, *n* 58).

Citric acid^456^ intensity for men, *r*_s_ 0·313, *P* = 0·034, *n* 36.

Notably, PROP scores did not correlate with intensity or hedonics of quinine HCl, nor with any of the test, dietary or demographic measures.

#### 6-*n*-Propylthiouracil taster type

There were 10–12 % non-tasters in each group (unlike the 30 % reported for the USA, but consistent with the 3–40 % reported worldwide^(^^16^^,^^22^^)^), resulting in samples too small for reliable analysis by group. There was no significant influence on any of the dietary and test variables, which did not differ by sex.

### Fungiform papilla density, taste intensity and hedonics

MANCOVA for two groups (EI were not assessed) and sex showed no significant effects, Nevertheless, with sex omitted, lingual fungiform papillae density tended to be lower in ES than NS (*F*(1,98) = 4·8; *P* = 0·031) (Table 1).

There were no significant correlations of papilla density by group or sex, with overall, or individual, basic taste psychophysics, intensity^456^, or hedonics^456^. There were significant correlations in specific subgroups. For salt^456^, ES showed a correlation with hedonics (*r*_s_ 0·483, *P* = 0·009, *n* 28), and for ES women, inverse with intensity (*r*_s_ −0·596, *P* = 0·007, *n* 19). For citric acid, women showed inverse correlations with hedonics (*r*_s_ −0·317, *P* = 0·018, *n* 55). For quinine^456^ hedonics, there was a correlation for NS men (*r*_s_ 0·585, *P* = 0·007, *n* 20). There were no significant correlations with sucrose^456^.

## Discussion

Across two immigrant and one native group of young people, graded by increasing familiarity with the dominant diet, the major dietary difference is increased intake of energy, fat, and, most prominently, Na^+^. Also trending from EI, to ES, to NS, were decreasing PROP responses, and hedonics of salt, sucrose and citric acid (intensity roughly following hedonics inversely), whereas adding salt, and eating tidbits in the laboratory, and anterior lingual fungiform papilla density increased. Together, these changes could reflect dietary acculturation, increasing overall intake, Na^+^ in particular, accompanied by decreasing taste sensitivity, and changes in sensory perception and preference in these Ethiopian immigrants.

### New immigrant diet and sodium appetite

The new immigrant (EI) diet in the immigrant centre comprised dinner prepared and served in the cafeteria, standard Israeli fare of the time. Immigrants prepared breakfast and lunch daily, and weekend dinner, in their apartments from bought food for which they received an allowance. They reported that 7·4 % of the foods they ate were purely Ethiopian, but some of the foods are common to both Ethiopian and Israeli diets, such as cooked vegetables and meat, fruit, etc. Therefore, EI dietary differences from ES and NS are probably conservative in comparison with the original Ethiopian diet^(^^40^^)^. More interestingly, their choice of foods within these constraints showed up as the difference from the other groups, most notably lower dietary Na^+^ intake. This is supported by EI liking scores, where Ethiopian staples scored high (all eight of twenty-four most liked foods), whereas common Israeli foods, such as pizza and ice-cream, scored low, suggesting preference guided their choices. Only in EI was there was a robust inverse correlation between the Na^+^ content of food items and liking scores, a salt adding score less than half of the other groups (Table 2), and only one EI adding ‘much’ salt compared with over 40 % of ES and NS.

### Sodium appetite

Dietary Na^+^ intake in the sample was relatively high, but similar to other groups we have studied^(^^29^^,^ ^30^^,^ ^32^^,^ ^34^^)^. Intake of Na^+^ is not a unitary phenomenon; it comprises untasted Na^+^ content of food, salting of food, which is partially a motor habit, preference for salty snacks, preference for salty foods, etc.^(^^32^^,^ ^41^^,^ ^42^^)^. Thus, EI show an inverse correlation between liking and Na^+^ content of food items, have a lower dietary Na^+^ intake, salt less than half of the other groups. However, in seeming contradiction, EI scored increased salt hedonics. In the tests they did not differ eating salty tidbits, or salting the soup, but overall, Na^+^ appetite progressed with acculturation. The dissociation between salt preference and intake is well documented^(14, 18, 23, 25, 32)^. Indeed, Ethiopians season food during preparation, not at table, consistent with the low EI salting we report, yet an Ethiopian saying illuminates their love of salt: ‘a person who does not behave appropriately according to practice, is like food without salt’^(^^43^^)^.

Finally, the marked progression of Na^+^ appetite with dietary acculturation seems specific, insofar as sweet preference does not differ.

### Taste sensory differences

PROP responses differ by group, which were highest in EI and lowest in NS (Table 1), were higher in women, and, particularly in the immigrant groups, correlate with intensity of oral sprays of salt, sucrose and, in men, citric acid and hedonics of sucrose. Notably, there is no correlation with the bitter taste of quinine HCl^(^^24^^,^^44^^,^^45^^)^. These responses in part are consistent with previous reports of higher scores in women, and correlations with intensity or hedonics of salt and sucrose^(^^16^^,^^32^^,^^38^^)^.

Lingual fungiform papilla density was not examined in EI, for which the procedure was too invasive. Papilla density was higher in NS than ES, and did not relate consistently to any of the measures, although subgroup analyses suggested that papilla density correlates inversely with intensity of salt, citric acid and quinine, particularly in women.

No relationship was found between PROP and papilla density, and neither of these predicted salt appetite, dietary intake, BMI, or any of the other tests and measures, broadly in line with variability in PROP response, and the probably marginal contribution to intake^(^^14^^,^^22^^–^^25^^)^.

The overall tendency for PROP responses and papilla density to be progressive from EI and ES to NS could suggest changes in taste perception over increasing exposure to native diet. In turn, this raises the possibility of changes with dietary acculturation in sensory neural and (epi)genetic substrates in papilla^(^^38^^,^^46^^)^ and, intriguingly, PROP response. Differences in PROP response with overall diet have been reported previously (although the authors favoured the opposite causality)^(^^13^^)^. An altered microbiome may also contribute to changes in papilla function^(^^47^^)^.

Categorisation of PROP responses into taster type provided no additional useful results.

### BMI

New immigrants had the lowest BMI (age-adjusted) with similar progression through the groups as repeatedly reported (Table 1)^(^^2^^,^^7^^,^^48^^)^. NS men differed from ES and EI men (24·7 (se 0·7), 21·7 (se 1·1), 20·0 (se 0·9); *P* = 0·015 and 0·000, respectively), whereas women did not differ by group. The data contrast with the alarming data on obesity and ill-health in this community, even in similarly 10- to 15-year veteran Ethiopian immigrants^(^^8^^,^^11^^)^, possibly because our sample was young and quite homogeneous.

### Sex differences

Some psychophysical responses and some taste sensory variables differed by sex. Primarily, women rated quinine as more intense, and were more averse to the tastes, particularly for citric acid and quinine, with a similar tendency for salt^(^^38^^,^^49^^)^. Their hedonic response appears concentration-dependent, whereas men tended to flatter curves, suggesting poorer discrimination of concentration (Figs 2 and 4).

PROP response was greater in women, but not papillae density. PROP in men predicted intensity of citric acid, and sucrose hedonics. Sex and group interactions for PROP differed by taste, hedonics and intensity, but despite some correlations, no clear pattern was discernable, consistent with the diverse results of other studies^(^^16^^,^^24^^,^^45^^)^.

Women sweetened their tea more, and reported lower alcohol intake and less activity. We found no relationship with week of menstrual cycle (data not shown)^(^^49^^)^.

### Limitations

Group sizes for EI and ES were determined by availability, and criteria of age, and time since immigration, although power was adequate (above), and analyses comparing Ethiopians as a group with NS (*n* 50 and *n* 82) confirmed the differences.

The cross-sectional design limited inferences about temporal processes, but comparisons at a single time-point excluded temporal changes in diet due to changing food preferences, trends, price, availability, etc. Cross-sectional comparisons of urbanisation within countries of origin revealed similar patterns of dietary acculturation and health consequences, supporting the suggestion that dietary differences may reflect acculturation, and length of residence has been suggested to reflect the sum total of an immigrant's experiences and exposures in their host society that make an impact on health^(^^50^^,^^51^^)^.

Whether differences relate to immigration or ethnicity cannot be determined from our data; however, in Ethiopia, the Jewish community was circumscribed in diet, region, ethnicity and religion, making it unlikely that EI and ES differed substantially in origin^(^^40^^,^^43^^)^.

Dietary questionnaires are of limited validity, as is the assessment of Na^+^ by any means^(^^52^^)^. Nevertheless, used to compare groups, as in the present study, absolute values are a lesser concern.

### Conclusions

Our findings confirm the increase in intake during dietary acculturation of immigrants to more affluent environments. More than previous studies, our results indicate that increased salt appetite may be prominent in dietary acculturation, as it is for Ethiopian immigrants to Israel. While diet changes may alter taste responses and psychophysics^(^^14^^,^^39^^,^^46^^,^^48^^,^^53^^)^, the finding that fungiform lingual papillae density, and the genetic response to PROP are altered, is novel.

The study adds Ethiopians to the immigrant community diets most often studied^(^^1^^,^^3^^)^. Ethiopian immigrant communities total some 1·5 million, over half in the USA, Saudi Arabia and Israel, the rest in Canada, Europe, Australia and Ethiopia's neighbours, but Ethiopian dietary acculturation has been sparsely studied^(^^11^^,^^12^^,^^40^^,^^54^^–^^57^^)^.

Understanding dietary acculturation is of interest for many reasons, among them the opportunity to improve healthy eating among immigrants, *inter alia* by maintaining the healthy aspects of their traditional diets (for Ethiopians – less fat and Na^+^), helping them anticipate the process and challenge of acculturation, and their vulnerability to the increased disease burden of diets of affluence^(^^4^^–^^8^^,^^10^^–^^12^^)^. Here we show that Na^+^ intake is greatly increased, which is useful to focus dietary advice, because excess Na^+^ may contribute to the health ailments afflicting immigrants^(^^1^^)^.

In practice, these immigrants’ nutrition is healthier in its low fat and Na^+^, suggesting that nutritional advice should focus on preservation, as well as prevention, and it may also enrich health and variety in the dominant diet^(^^6^^,^^8^^)^. Other useful insights may also be gained. For example, the fact that these immigrants find salt more hedonic but eat less of it, whereas natives find it less hedonic but eat more of it, might suggest that the high native Na^+^ intake may be due to reduced sensitivity to the taste of Na^+^, which may be restored^(^^12^^,^^13^^,^^46^^)^. Similar taste sensory adaptations to changing diet may occur during dieting, or after bariatric surgery, etc.^(^^12^^,^^13^^,^^46^^,^^53^^)^.

Such knowledge can guide preferences toward a healthier diet for all.
